# A novel device-assisted endoscopic ultrasonography-guided single balloon-occluded gastrojejunostomy bypass with a three-dimensional-printed three-way connector

**DOI:** 10.1055/a-2930-8760

**Published:** 2026-10-01

**Authors:** Ping Ping Zhang, Yu Chen Qian, Kai Xuan Wang

**Affiliations:** 1 Department of Gastroenterology 12520 Changhai Hospital, Naval Military Medical UniversityShanghaiChina; 2 Department of Gastroenterology 12520 Changhai HospitalShanghaiChina

## List of abbreviations

EUS: Endoscopic ultrsonography
CT: Computed tomography
LAMS: Lumen-apposing metal stents

A 58-year-old man presented with a 2-month history of vomiting. Laboratory tests showed elevated carbohydrate antigen 19-9. Contrast-enhanced computed tomography revealed pancreatic cancer with the invasion of the horizontal duodenum. Gastro-enterography showed duodenal narrowing ( Fig. 1 ).

**Fig. 1:**
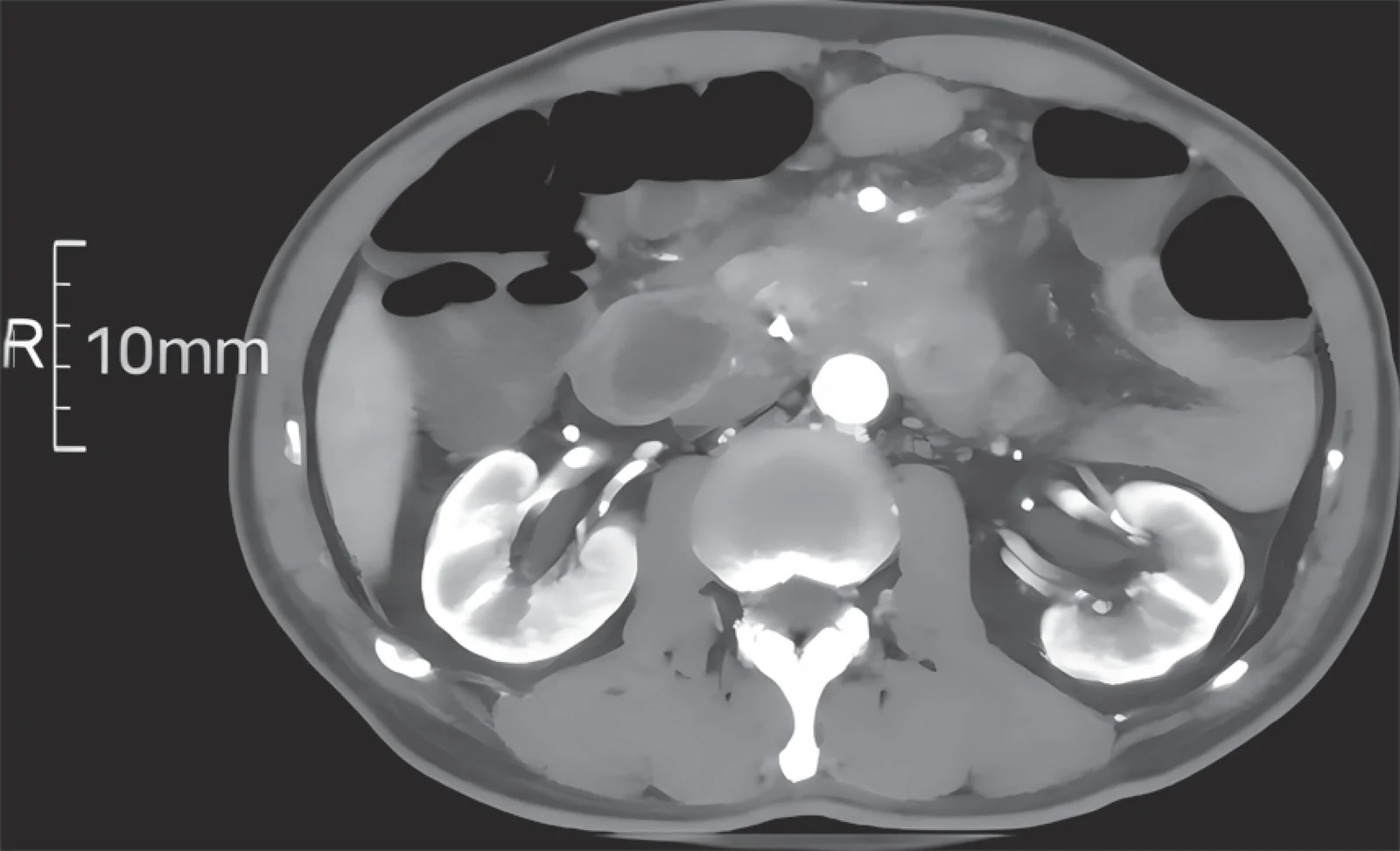
Contrast-enhanced CT revealed pancreatic cancer with the invasion of the horizontal duodenum, and gastro-enterography showed duodenal narrowing.

We developed a novel device-assisted Endoscopic ultrsonography (EUS)-guided single balloon-occluded gastrojejunostomy bypass. The device consists of a catheter assembly and a 3D-printed three-way connector shaped like an aircraft tail ( Fig. 2 ). After connecting the connector, inject 40 mL of air through the balloon port to achieve a 4 cm balloon diameter. Once the connector is removed, the balloon remains briefly inflated, maintaining its position during device exchange.

**Fig. 2:**
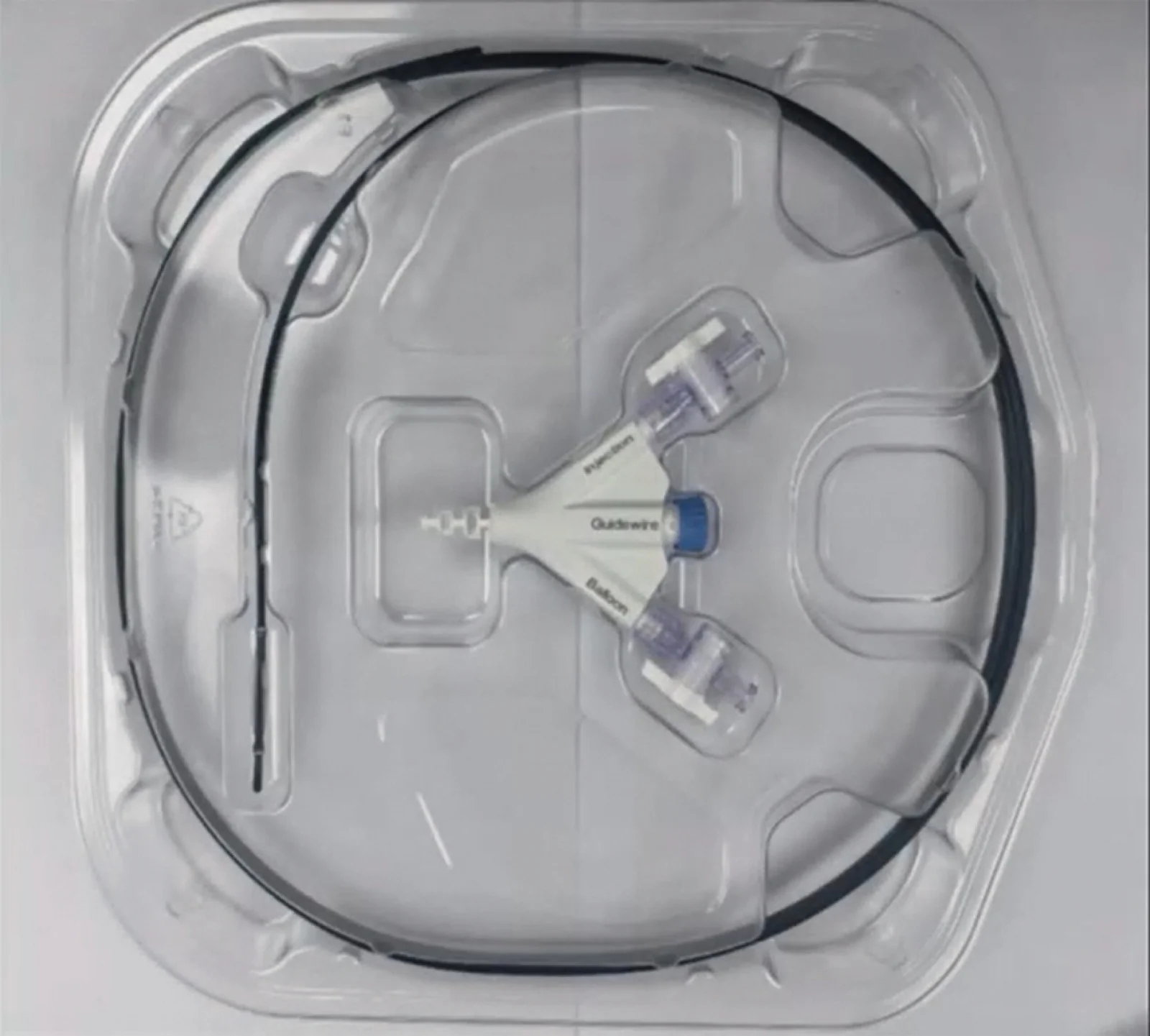
The device consists of a catheter assembly and a 3D-printed three-way connector shaped like an aircraft tail.

Under fluoroscopy, a contrast catheter and a guidewire are advanced through the endoscope’s accessory channel, crossing the obstruction into the distal jejunum. The balloon catheter is advanced over the guidewire, and air is instilled to occlude the lumen. The endoscope is withdrawn while the catheter remains in place, and the connector is reconnected. A mixture of saline, contrast, and methylene blue is injected. A curved linear echoendoscope identifies the fluid-filled distal duodenum and proximal jejunum adjacent to the gastric body. Under EUS guidance, a lumen-apposing metal stent is deployed through the gastric and jejunal walls using an electrocautery-enhanced delivery system. Methylene blue-stained fluid flowing into the stent confirms the correct positioning of the distal flange within the jejunum ( Video 1 ).

**Video 1** The procedural process of the novel device-assisted EUS-gastrojejunostomy performed on the patient.


Endoscopy_UCTN_Code_TTT_1AS_2AK

